# Access to Care Limits Lung Cancer Screening Eligibility in an Urban Safety Net Hospital

**DOI:** 10.1177/21501319221128701

**Published:** 2022-10-06

**Authors:** Krista R. Dollar, Bradley S. Neutel, David W. Hsia

**Affiliations:** 1 Harbor-University of California Los Angeles Medical Center, Torrance, CA, USA; 2 The Lundquist Institute for Biomedical Innovation at Harbor-UCLA Medical Center, Torrance, CA, USA

**Keywords:** access to care, lung cancer screening, prevention, underserved communities, primary care

## Abstract

**Purpose:**

Lung cancer screening (LCS) results in earlier detection of malignancy and decreases mortality but requires access to care to benefit. We assessed factors associated with timing of lung cancer diagnosis in the absence of systematic LCS in an urban safety net hospital.

**Patients and Methods:**

Retrospective chart review was performed of patients with pathologic diagnosis and/or staging of lung cancer at our institution between 2015 and 2018. Patient socio-demographics, disease characteristics, factors associated with access to medical care, and time point and process by which the patient accessed care were collected and analyzed.

**Results:**

In total, 223 patients were identified with median age of 63 years and 57.8% male predominance. Racial distribution was 22.9%, 20.2%, 17.1%, and 9.4% for Black, White, Asian, and Hispanic, respectively. Stage at diagnosis was 8.1%, 4.5%, 17.0%, and 60.5% for stages I, II, III, and IV, respectively. Medicaid (59.6%) and Medicare/Medicaid (17.1%) were the most common insurance types, while 16.1% had no insurance. A majority (54.3%) had no established primary care provider (PCP), and only 17.9% had an in-network PCP. Patients without PCPs were more likely to have diagnostic evaluation initiated from Emergency Department or Urgent Care settings (95.0% vs 50.1%, *P* < .01) and present with later stage disease (92.7% vs 77.8%, *P* < .01). Of the 83 patients that met age and smoking history LCS criteria, only 33.7% (12.6% of total) also had an in-network PCP.

**Conclusion:**

Absence of established PCPs is associated with later stage presentation of lung cancer and may limit system- level benefits of LCS implementation.

## Introduction

Lung malignancy is the leading cause of cancer-related death, accounting for approximately 1 in 5 deaths; in the United States alone, there are an estimated 236 000 new lung cancer cases and 132 000 lung cancer-related deaths annually.^1^ Stage at diagnosis affects available treatment options and survival, making early detection important in the management of the disease. Patients diagnosed with localized non-small cell lung cancer (Stage I) have a 57.4% 5-year survival which contrasts with 5.2% for those diagnosed with metastatic disease (Stage IV).^1^

In 2013, the United States Preventive Services Task Force (USPSTF) recommended annual lung cancer screening (LCS) for asymptomatic persons aged 55 to 80 years with a smoking history of at least 30 pack years who are currently smoking or quit within 15 years.^2^ This recommendation was largely based on data from the National Lung Screening Trial (NLST) which showed a 20% relative reduction in lung cancer mortality after 3 years of screening with low- dose computerized tomography (LDCT) compared with chest X-rays in high-risk patients.^3^ Subsequently, the Dutch-Belgian Randomized Lung Cancer Screening Trial (NELSON) reported a 24% lower risk of lung cancer-related deaths in high-risk patients randomized to receive low-dose computerized tomography (LDCT) compared to no screening at 10-year of follow-up.^4^ Despite this demonstrated benefit, LCS uptake has been slow and variable across the United States.^5^ In 2016, only 3.3% of the estimated 8 million eligible patients in the United States underwent screening. On par with these results, Jemal et al found that screening remained comparably low in 2015 versus 2010 despite the interval introduction of USPSTF screening recommendations in 2013.^6^ Furthermore, geographic variation persists in regards to LCS. LCS centers are largely clustered in urban areas causing variability in availability of LCS within the same region.^5^

In addition to variability in availability of LCS, there are racial differences in both smoking behaviors and age at diagnosis that likely contribute to racial disparities in lung cancer detection using uniform screening eligibility.^7^ These differences in behaviors and disease result in varying performance characteristics of the NLST criteria notably in minorities prompting broadening of USPSTF guidelines in 2021 to ages 50 to 80 and a 20 pack year smoking history.^8^

LCS is initiated at the primary care provider (PCP) level with patient education and shared decision making about the implications of abnormal findings on LDCT. Implementing a LCS program also requires institutional infrastructure and support, including an interdisciplinary team, standardized reporting systems, protocols for patient follow-up, and strategies to provide effective patient counseling and shared decision making.^9^ These systematized processes represent a large commitment of institutional resources, such as equipment, personnel, and education of patients and medical providers which can be an institution-level barrier to LCS. In addition, there are numerous factors that may influence a patient’s access to medical care. The complex interaction between these factors and other determinants of health is highly individualized making it challenging to understand how different patient populations at institutions are affected. Prior evaluations of barriers to medical care in LCS populations have been from high-risk screening patients already within a medical system. However, these descriptions do not provide insight into the magnitude that this problem represents for those who have not yet established continuity care with a PCP who ultimately develop lung cancer and would benefit the most from access to LCS.

Our institution is a key provider of inpatient and outpatient services within a large urban safety-net health care system serving a diverse and largely underserved patient population. The purpose of this exploratory study is to describe the population of patients diagnosed with lung cancer at our institution who differ considerably from LCS study populations and assess how demographic, socioeconomic, and access to health care might influence the diagnosis of lung cancer in the absence of a systematic LCS program. One specific objective is to assess the percentage of lung cancer patients that would qualify for LCS. Secondly, we aim to quantify the impact that lack of access to care has on LCS by determining the number of lung cancer patients who did not have routine medical prior to diagnosis but would otherwise meet LCS eligibility criteria. There are numerous reports of LCS utilization amongst high-risk populations of patients who already have established medical care within a healthcare system. However, quantitation of the number of patients who do not have access to medical care prior to lung cancer diagnosis is not well described in the medical literature. Quantifying the magnitude of the barrier represented by access to care further provides perspective on the percentage of lung cancer patients within the patient population that might benefit from LCS.

## Methods

Retrospective chart review was performed on all patients undergoing diagnostic or staging procedures with a new diagnosis of primary lung cancer at our institution from 2015 to 2018 at Harbor-UCLA Medical Center (Torrance, CA). Patients were identified from a hospital database of cancer patients. All patients with pathologic diagnosis or staging of cancer were included for analysis. Data was retrospectively collected from the electronic medical record, which includes records from all levels of care within the Los Angeles County-Department of Health Services (LAC-DHS) system including primary care providers, subspecialty clinics, urgent care, and the emergency department. Socio-demographic data including insurance status and primary language, smoking status, and characteristics of lung cancer and medical care including diagnostic workup and treatment were recorded in de-identified fashion. Patients who obtained care from a primary continuity care setting within our provider network were classified as having an in-network PCP versus those who received continuity medical care externally who were classified as having an out-of-network PCP. Assessment of an established PCP at least 12 months prior to cancer diagnosis was identified by presence of documentation in the electronic medical record by a medical provider, indication of a PCP on hospital intake forms or in medical documentation including outside medical records, or indication of routine access to prescription medications or other recurrent medical care. Insurance status was differentiated as public insurance from national (Medicare) and state (Medicaid) levels versus private insurance provided by third party sources.

Descriptive analysis was performed with median and interquartile range (IQR). Lung cancer staging was performed using the Eighth Edition of the TNM Classification system.^10^ Qualification for LCS was determined using the 2013 and 2021 USPSTF lung cancer screening guidelines.^2,8^ Distance to the medical center was calculated using Google Maps. Associations between variables was performed using Fisher’s exact test with a threshold of *P* < .05 used to determine statistical significance. This study was granted an exempt determination by The Lundquist Institute Institutional Review Board.

## Results

A total of 223 newly diagnosed or staged lung cancer patients were identified during the target time period. Median age at diagnosis was 63 with an IQR of 55 to 69 and a male predominance (Table 1). Of the patients analyzed, 71.7% ranged in ages from 50 to 80. The largest racial group was Black followed by White. The majority of patients were either current or former smokers with 41.3% having at least 20 pack years of smoking and quit <15 years prior.

**Table 1.** Demographics of All Lung Cancer Patients Further Divided by Ethnicity.

	All patients	Asian	Black	Hispanic	White	Other
Number (%)	223 (100)	38 (17.0)	51 (22.9)	21 (9.4)	45 (20.2)	68 (30.5)
Age						
Median [IQR]	63 [55-69]	65.5 [58.25-68.75]	61 [54-66.5]	62 [54-71]	63 [58-70]	61.5 [53-69]
Pt. ages 50-80 (%)	160 (71.7)	33 (86.8)	39 (76.4)	16 (76.2)	39 (86.7)	54 (79.4)
Sex						
Male (%)	129 (57.8)	20 (52.6)	23 (45.1)	16 (76.2)	26 (57.8)	44 (64.7)
Female (%)	94 (42.2)	18 (47.4)	28 (54.9)	5 ( 23.8)	19 (42.2)	24 (35.3)
Smoking history						
Current or former(%)	157 (70.4)	23 (60.5)	44 (86.3)	13 (61.9)	33 (73.3)	45 (66.2)
Never (%)	66 (29.6)	15 (39.5)	7 (13.7)	8 (38.1)	12 (26.7)	23 (33.8)
Pack years						
>20 (%)	113 (71.9)	14 (36.8)	24 (47.0)	11 (52.3)	28 (62.2)	35 (51.4)
<20 (%)	30 (19.1)	7 (18.4)	12 (23.5)	2 (9.5)	2 (4.4)	7 (10.3)
Not Quantified (%)	14 (8.9)	2 (5.2)	8 (15.7)	0 (0)	3 (6.6)	3 (4.4)
>20 and quit <15 years ago (%)	92 (41.3)	10 (26.3)	21 (41.2)	8 (38.1)	24 (53.3)	30 (44.1)

The majority of patients were stage IV at time of diagnosis. Distribution of disease stage were 8.1%, 4.5%, 17.0%, and 60.5% for stages I, II, III, and IV, respectively. The most common types of lung cancer were adenocarcinoma (48.4%), squamous cell carcinoma (18.8%), small cell carcinoma (9.9%), and mixed type (7.6%). Genetic driver mutations were identified in 59 (26.5%) patients, though testing was not applicable in 90 (40.3%) based on cancer subtype or stage.

With regards to distance to the medical center, 24.7% lived within a five-mile radius, 40.3% lived between 6 and 10 mi, and 35.0% lived more than 10 miles away. At time of diagnosis, 7 (3.2%) patients did not have stable housing. Any type of medical insurance was identified in 187 (83.9%), with Medicaid alone (n = 133, 59.6%) and Medicare/Medicaid (n = 38, 17.1%) being the most common insurance types; 2 (0.9%) had private insurance while 36 (16.1%) had no health insurance. Only 102 (45.7%) had an established PCP at least 1 year prior to diagnosis, of which 63 (28.2%) had a PCP within our medical network.

**Figure 1.**
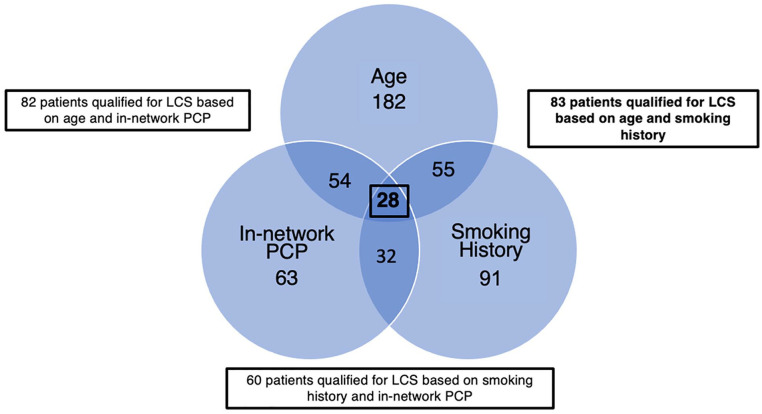
Patient eligibility for lung cancer screening based on age (50-80 years), smoking history (greater than 20 pack years and current smoker or quit ≤15 years ago) and established in-network primary care provider, n = 223 patients.

Using 2013 USPSTF screening guidelines which were clinically applicable during the study time frame, 61 (27.3%) patients qualified for LCS based on combination of age and smoking history criteria. Of those patients meeting 2013 USPSTF LCS eligibility criteria, only 26 (42.6% of LCS eligible patients) had an established PCP within our healthcare system. When applying updated 2021 USPSTF screening guidelines to the study population, 83 (37.2%) patients met LCS eligibility criteria (Figure 1) of which 28 (33.7% of LCS eligible patients) had an established in-network PCP.

Early cancer presentation (stage I/II) occurred in 20 out of 90 (22.2%) with an established PCP and 8 out of 110 (7.3%) without an established PCP (*P* < .01) (Table 2). For patients with health insurance coverage, 22 of 164 (13.4%) were diagnosed at early stage compared to 6 of 36 (16.7%) with no identified insurance (*P* = .61). Twenty-three patients lacked a confirmed stage at diagnosis and were not included in the analysis.

**Table 2.** Early Stage Disease Versus Late Stage Disease Based on established PCP Status and Insurance Status.

	Stage I/II	Stage III/IV	*P* value
Established PCP	20	70	<.01
No established PCP	8	102	
Any insurance	22	142	.61
No insurance	6	30	

Diagnostic evaluation was initiated due to cancer-related symptoms in 173 (77.6%), CT screening by the patient’s PCP in 8 (3.6%), and incidental findings on chest imaging performed for other clinical reasons in 42 (18.8%) (Table 3). The locations of initial medical evaluation and referral for diagnostic workup that eventually confirmed lung cancer were the emergency department (70.4%), primary care clinic (13.0%), and subspecialty clinic (11.2%). Those without an established PCP were more likely to have diagnostic workup initiated through the emergency department or urgent care than those who had an established PCP (95.0% vs 51.0%, *P* < .01) (Table 4).

**Table 3.** Workup and Referral Site by Established PCP Status.

	All patients (n = 223) (%)	Established PCP (n = 102) (%)	No established PCP (n = 121) (%)
Workup			
Symptomatic	173 (77.6)	75 (73.5)	98 (81.0)
Incidental Finding	42 (18.8)	19 (18.6)	23 (19.0)
CT screening by PCP	8 (3.6)	8 (7.8)	—
Referral site			
Emergency department	157 (70.4)	45 (44.1)	112 (92.6)
Urgent care	10 (4.5)	7 (6.9)	3 (2.5)
Primary care clinic	29 (13.0)	29 (28.4)	0
Subspecialty clinic	25 (11.2)	19 (18.6)	6 (4.9)
Unknown	2 (0.9)	2 (20.0)	0

**Table 4.** Referral Site Initiating Diagnostic Workup Based on Established PCP Status.

	Emergency room or urgent care	Clinic	*P*-value
Established PCP	52	50	<.01
No established PCP	115	6	

Regarding components of cancer-related care received within our medical system, 147 (65.9%) received all of their care from clinical presentation to initial diagnostic procedure to treatment in our health care system. Forty (17.9%) patients were diagnosed outside of our medical system but completed staging and subsequently established medical care for cancer treatment at our hospital. Thirty-six (16.2%) patients received subsequent medical care outside our medical network after initial diagnosis (13.5%) or staging (2.7%).

## Discussion

Lung and bronchus cancers account for the most cancer-related annual deaths, with a 5-year relative survival of 21.7%.^11^ Lung cancer screening with LDCT has been shown to effectively reduce lung cancer mortality.^4^ Despite this, implementation of LCS remains low and variable across different populations. Only 5% of high-risk adults underwent screening based on national data from 2015, which is comparable to the 3.3% and 3.4% estimated national screening rates in 2016 and 2017, respectively, which were drawn from comparison of radiology registry data to population-based surveys, the U.S. Census, and cancer registry data.^5,12-15^ There was slight improvement in 2018 to 5.0%, however geographic variation persists. Other multi-state samples from 2017 and 2018, demonstrated approximately 15 to 20% of eligible high-risk patients receiving LCS, however again demonstrating significant variability by region and insurance status.^12-15^ In fact, California had the second lowest LCS rate (of eligible patients) with a 0.21 screening rate ratio compared to the national average.^5^ LCS sites remain clustered in urban areas and more than a third of the counties with high lung cancer mortality were beyond a 60 min drive from a LCS center.^16^

There are a multitude of different barriers to LCS that have been identified and can be broadly categorized as patient-, provider-, and system-level barriers.^17^ Much conversation in the medical literature focuses on provider- and system-level barriers to implementation of LCS. One study found that providers practicing at a community or academic hospital order LDCT scans more frequently than those practicing at a safety net hospital.^18^ Provider knowledge of LCS guidelines varies and suggests the need for improved provider education at safety net hospitals.^18^

Our institution is a tertiary referral center within LAC-DHS providing subspecialty care for the diagnosis and treatment of lung cancer with a catchment area covering the southern portion of the County of Los Angeles. Our patient population shares similarities in many characteristics of disease to national data. The sociodemographic diversity demonstrated in our patient population highlights the challenges of other medical systems and providers across the nation. The NLST study population was over 90% White but lung cancer behaves differently amongst different demographic groups. Therefore, performance of screening guideline eligibility does not align equally for Blacks and Whites.^7^ In the NLST, Black patients had greater reduction in both lung cancer and all-cause mortality compared to Whites, even with low participation.^3^ Despite having greater lung cancer incidence, Blacks are less likely to be eligible for screening given their lower average cigarette per day consumption and are nearly half as likely to report undergoing LCS than their White counterparts.^7,19^ It is possible that with a lower pack year smoking history there will be an increase in the proportion of eligible Black patients,^7^ however even the updated 2021 USPSTF guidelines do not take into account racial, ethnic, or socioeconomic differences in smoking patterns and lung cancer risk and may not be optimal for high risk populations like underrepresented minorities.^19^

Nationally, lung cancer is most prevalent amongst patients age 65 to 74. Our patients were diagnosed younger, but still skewed toward stage IV metastatic disease.^1,11^ The racial distribution in our population was significantly more diverse, with greater Black, Asian, and Hispanic representation.^11^ Though only 9.4% of our patients were Hispanic, we suspect that this is an underrepresentation and limitation of the retrospective nature of the data collection as 32.7% listed their preferred primary language as Spanish. Therefore, it is probable that a significant contingent of the 30.5% of patients listed within the Other racial category are of Hispanic origin. Presentation of lung cancer differs by race with earlier onset (median age 67 vs 70 years) and more advanced stage disease (53% vs 49%) being found in Blacks versus Whites and could contribute to the observed differences.^20,21^

Cigarette smoking has been attributed to 81% of deaths in lung, bronchus and tracheal cancers and is mirrored in the high prevalence of tobacco use in our population.^22^ Nevertheless, almost 30% of our patients were never smokers which may be associated with the high prevalence (44.4%) of genetic driver mutations identified in those who were clinically appropriate for testing. Prevalence of driver mutations varies widely, however, many that are associated with earlier development of lung cancer are more common in certain demographic groups such as Asians and non- or light-smokers.^23,24^

Disparities in lung cancer incidence, diagnosis, treatment and mortality are well documented in the medical literature and are associated with demographic and socio-economic groups.^20,25^ Patients with low socioeconomic status are more likely to have medical insurance-related limitations resulting in lack of access to LCS, leaving this vulnerable population at increased risk for lung cancer without an equitable way to access screening opportunities.^26^

These disparities can often be associated with a complex interaction of variables, such as communication, education and medical literacy, and factors associated with access to care, such as proximity to the clinic or hospital. In our patients, 45.7% had a primary language other than English, with 32.7% of patients being Spanish speaking. The presence of a language barrier creates extra challenges in health literacy as medical explanations can be lost in translation despite the use of interpreters resulting in miscommunications. Housing and proximity represent additional barriers; 3.1% of our patients were unhoused at time of diagnosis, though this likely is an underrepresentation due to the retrospective nature of the study data collection. While 24.7% of patients live within 5 mi of the hospital and 65.0% of patients live within 10 mi of the hospital, 35.0% live more than 10 miles away. Proximity is a recognized indicator of access to care, however other important considerations such as the mode and access to transportation along with the time requirement for commuting to the medical center could not be accurately assessed from this retrospective chart review and would offer more insight into our patients’ barriers to care.^27,28^ Other studies note that drive time is significant for some patients secondary to the geographic variability of LCS locations, however the method of transportation is not assessed.^16^ Patients also face an opportunity cost when obtaining medical care due to other social and financial considerations such as family or work responsibilities but this information is not represented in the available data. Nevertheless, these data represent social determinants of health that may play a role in accessing medical care for patients. Access to care is a challenging issue and it is likely that complex interactions between various factors confounds the ability to adequately describe it in terms of individual variables with our data set.

Medical insurance status is another recognized marker of access to care and has been associated with improved lung cancer survival for patients with private health insurance compared to those with Medicaid or no insurance.^29^ In our study, 16.1% of patients had no identifiable insurance, 59.6% had Medicaid alone, 83.0% of patients had only public insurance (Medicare and/or Medicaid), and 0.9% had private insurance. This contrasts with national data from 2018 indicating that private insurance coverage (67.3%) is substantially more prevalent than public coverage (34.4%) in the United States.^30^ LCS coverage by Medicaid is determined at the state level causing wider variation in coverage.^31^ Until recently, Medicaid was one of the only healthcare payer programs not required to cover LCS.^32^ Increased prevalence of severe comorbidities in this older patient population may result in fewer recommendations for LCS by PCPs.^18^ We did not find a significant association between lack of medical insurance with diagnosis at an earlier stage of disease though other outcome variables such as mortality were not assessed. Even though there was no demonstrated relationship between insurance status and stage of disease in our cohort, it is probable that the study population size and complex interaction with other socioeconomic and demographic factors may limit our ability to detect this association. Lack of medical insurance or housing are likely to be higher than identified as patients qualify for public insurance once diagnosed with cancer. Therefore, when retrospectively collected, status of these variables may not be reflective of the time of diagnosis unless explicitly indicated in the medical record.

Absence of an established PCP was the most pronounced obstacle to allowing our patients to participate in potential LCS. When considering having any established PCP regardless of in-network versus out-of-network status as a gateway to potential LCS access, 121 (54.3%) did not have an established PCP 1 year prior to diagnosis and therefore would not typically have access to routine health maintenance. There was a significant difference in patients presenting with earlier stage disease who had an established PCP as compared to no established PCP (*P* < .01). This may be influenced by a variety of factors beyond LCS, such as incidental pulmonary findings from workup of other issues, differences in how patients approach their health and medical care amongst those who have established access to care, or social/cultural perspectives regarding medical care.

Out of our total population, 27.4% qualified for LCS based on age and smoking history criteria using the 2013 USPSTF guidelines but 57.4% of eligible patients did not have a PCP within our medical system. Therefore only 42.6% of eligible patients would have benefited from a screening program at our institution with the remainder of eligible patients unable to access LCS without a PCP. Using the 2021 USPSTF guidelines expands eligibility to 37.2% of all patients qualifying for LCS, but only 33.8% of LCS eligible patients had an established in-network PCP. In addition, it is worthwhile to note that expanded 2021 USPSTF LCS eligibility criteria increases the number of eligible patients for screening by 36.1%, however this only translates to a 7.7% increase when accounting for access to care.

Despite the association between PCP status and earlier stages of disease, 73.5% of patients with an established PCP still presented symptomatic from disease. Lack of an established PCP has been associated with worse lung cancer mortality, and implementation of LCS would promote earlier identification of malignancy as well as initiation of workup from primary care settings.^33^ After diagnosis, 83.8% of patients stayed in network for continuity of treatment which demonstrates the reliance of our patient population upon our safety net medical center for their medical treatment.

LCS continues to be underutilized nationally, and studies at other safety net institutions report an overall estimated screening rate of only 16%.^25^ In a retrospective description from a single-center, Olazagasti et al reported that 35% of lung cancer patients qualified for LCS however only 4.8% of patients actually underwent screening. A significant association was also found between stage at diagnosis and screening with LDCT.^34^ To our knowledge, this is the only other study that describes the percentage of lung cancer patients that would qualify for LCS as all other data describe LCS utilization amongst in-network populations of patients who meet eligibility criteria. In addition, factors associated with access to care such as insurance type and geographic relationship to the medical facility have been associated with the utilization of LCS.^28^ However, the interplay of factors associated with access to care is complex and does not always directly equate to lack of medical care. Our data is unique in that it quantitates the percentage of LCS-eligible patients diagnosed with lung cancer who would not be able to undergo screening due to lack of access to medical care.

While access to care remains a significant barrier to LCS, this does not diminish the benefit of screening programs for established patients. However, our study highlights the challenge that access to medical care represents for underserved populations and how it may lead to disparities in lung cancer. In addition, the lack of continuity health care prior to lung cancer diagnosis coupled with a high retention rate for cancer-related care afterward also suggests that system-level benefits from implementation of LCS is likely to be reduced unless improvements can be made in establishing earlier access to care. It is important to note that even with medical insurance and access to care, LCS still is only performed in approximately 10% to 25% of eligible patients.^35^ Furthermore adherence to the program is also an issue; at a hospital serving a diverse population, annual follow-up was only 23.7% after 1 year and dropped to 2.8% after 2 years.^36^

Based on current USPSTF guidelines there is ongoing concern that multilevel barriers to implementation of LCS and uniform eligibility guidelines will not adequately address the disparities in lung cancer outcomes. Most societal screening criteria utilize age and smoking history similar to USPSTF guidelines. There are other proposed risk models that can be used aside from USPSTF guidelines that include factors beyond just age and smoking history, but improvements are still needed. These risk models are based on little data from minorities and thus still underestimate risk by 5% to 25% in these patients.^37^

There are several limitations that influence the interpretation of this data. This is a small patient cohort representing a single-center experience, thus limiting generalizability to other institutions or medical care environments. The smaller sample size also limits the evaluation of broader range factors that might affect access to care. Despite this, our medical center and associated clinics provides medical care to approximately one-third of all patients within our regional public safety net system. Therefore, these data provide a representative cross-section of the patients in our urban medical system. This was a retrospective study and all data were collected from a single evaluation of the medical record. Some variables are not discrete entries within the electronic medical record and therefore relied upon documentation within medical and ancillary provider notes. In addition, data in patient health records at time of chart review may have been subsequently updated and does not always reflect the situation at time of diagnosis, and may result in inaccuracies compared to other methods of data collection.^38,39^ Review of records for patients receiving components of care external to our medical system were limited to available data in our health records and documentation of patient self-reporting by medical providers.

Through this study we have identified several issues in a diverse safety net population, however further understanding the barriers that influence our population’s access to care is critical. To our knowledge this data is unique in that it quantifies the magnitude of the problem represented by lack of access to medical care and the impact it would have on availability of LCS to an underserved patient population. For the future, prospective assessment of high risk patients who recently established care is needed in order to elucidate their barriers to establishing care and difficulties to undergoing LCS. These factors may include medical literacy, cultural and social perceptions of medical care and lung cancer, financial ability to pay, or financial freedom to take time off work to obtain medical care. These prospective studies will provide valuable insights into underserved patients’ barriers to accessing care that are difficult to draw from a retrospective study. Based on this data, we will need to assess the value of implementing a LCS program that has a broader eligibility than the USPSTF 2021 guidelines. Risk models will need to be analyzed and evaluated when used in our underserved and diverse patient population given the performance variability in different patient demographics.^40^ Additional evaluations of various risk models need to be performed to avoid an underestimated risk of lung cancer in minorities to ultimately further reduce LCS disparities.

## Conclusion

Lack of a PCP in underserved and underrepresented urban patient populations is associated with later stage presentation of lung cancer and may limit system-level benefits of LCS implementation. Further efforts are needed to understand and overcome barriers in access to care to reduce disparities in lung cancer.
